# One-to-one peer-coaching for patients with cancer — results of a pilot study

**DOI:** 10.1007/s00432-024-05913-0

**Published:** 2024-08-07

**Authors:** Alice Valjanow, Joachim Weis

**Affiliations:** Comprehensive-Cancer Centre Freiburg (CCCF), Department of Self-Help Research University Clinic, Medical Faculty of the Albert-Ludwigs University, Hugstetterstr. 49, Freiburg, 79106 Germany

**Keywords:** Cancer, Empowerment, Patient participation, Peer-coaching, Psycho-oncology, Quality of life

## Abstract

**Purpose:**

Cancer is a life threatening disease with negative impact on quality of life and psychological well-being. In international studies, one-to-one peer support and counseling have been shown to improve the psychological well-being of cancer patients. In the study presented, we developed and evaluated an innovative program of peer-coaching. In this program at the University Hospital of Freiburg, cancer survivors were trained to support peers by sharing experience.

**Methods:**

In the project, *N* = 25 cancer survivors were trained to conduct supportive one-to-one conversations with acute patients or patients in aftercare. Based on a prospective observational study, patients were interviewed using questionnaires before and after the conversations. We assessed expectations and experiences with the peer-coaching as well as psychosocial parameters (PHQ9, GAD7, SSUK, NCCN-distress thermometer).

**Results:**

A total of 52 patients had at least one contact with a peer-coach. Most of the patients attended 1–3 sessions. In total, 85 contacts pairing peer-coaches with patients were conducted. Patients showed on average a high level of distress but a low rate of psychiatric comorbidity. The supportive conversations met the patients` needs. Sharing experiences and empowerment were the most relevant benefits for the patients. Both patients and trained peers showed high satisfaction levels with the program.

**Conclusion:**

Our findings support the feasibility and utility of a peer-coaching program in which trained cancer survivors, acting as peer-coaches, support other patients during or after their oncological treatment. In a further study, the efficacy of peer-coaching should be investigated based on a randomized-controlled trial.

**Trial registration:**

The trial was registered in the German Clinical Trials Register (No. DRKS DRKS00017500) on 12.12.2019.

## Introduction

Cancer is a critical life event with high incidence rates and a growing amount of survivors all over the world (World Cancer Research Fund International 2020). According to the Robert-Koch-Institute (RKI), nearly 500.000 people in Germany are newly diagnosed with cancer every year (Erdmann et al. 2021). The number of cancer patients in Germany is expected to increase from the year 2015 to 2030 by around 23% (Erdmann et al. 2021). Fortunately, improvements in the prevention, early detection and treatment of cancer in the last decades led also to increasing rates of longtime survivors (Soerjomataram et al. 2008). Both the growing number of patients living with or after cancer and the improved long-term survival rate lead to a greater need for psychosocial services that improve the cancer patient’s psychological well-being and quality of life (Foster et al. 2009). Cancer experience has the potential to be significantly distressing for patients (Bultz and Johansen 2011). There is a wide range of prevalence rates for psychological problems of long term survivors. In a systematic review of 20 studies, the reported prevalence of depressive symptoms varied from 5.4 to 49.0% (pooled prevalence: 21.0%), for anxiety from 3.4 to 43.0% (pooled prevalence: 21.0%) and for distress ranged from 4.3 to 11.6% (pooled prevalence: 7.0%) (Brandenbarg et al. 2019). In a register-based survey of more than 1000 German long-term survivors, 17% reported at least moderate depression symptoms and 9% reported anxiety symptoms (Götze et al. 2020). An analysis of the unmet needs of cancer patients revealed that there is a need for peer support in social, spiritual and practical domains and especially in the area of information (Park et al. 2019). Social support, such as peer support, has been identified as a key resource to buffer stressful experiences due to cancer (Allicock et al. 2014) and can lead to psychological empowerment among cancer patients (Ziegler et al. 2022). Peer support is defined as a non-professional support service for cancer patients provided by cancer survivors who have had a similar diagnosis or treatment situation. It includes psychosocial support based on shared personal experiences (Dunn et al. 2003). Peer support is considered an indispensable mental health service and is emerging as standard of practice with the potential to impact several clinical outcomes (Fortuna et al. 2022). First studies on the benefits of one-to-one peer-counseling in cancer patients showed positive effects on variables such as self-efficacy, feeling of control, self-management, active coping and knowledge, cancer related well-being, increases in hope and confidence as well as stimulation of healthy behavior and cancer specific quality of life (Allicock et al. 2014; Giese-Davis et al. 2016; Kiemen et al. 2023; Lee et al. 2013; Slesina et al. 2014; Weber et al. 2007; Zhang et al. 2022; Ziegler et al. 2022). In addition, studies found that peer support has the potential to reduce anxiety, emotional distress and depression and to diminish the feeling of social isolation (Macvean et al. 2008; Slesina et al. 2014; Ussher et al. 2006; Zhang et al. 2022). Not only the patients, but also the trained peers can benefit from participating in peer support programs, e.g. by sharing their own experiences, helping others or improving their own communication skills (Allicock et al. 2014). Thus, the peer-coaches may strengthen their own self-esteem and mental well-being or gain new perspectives on their previous cancer experience (Pistrang et al. 2013).

Despite promising findings in international studies, White et al. (2020) concluded in their systematic review and meta-analysis, that one-to-one peer support might have a positive impact on psychosocial outcomes of the patients but does not significantly improve clinical outcomes. In peer support groups, group members are rarely prepared for the supportive conversations with peers and are seldom accompanied by psychological professionals in case of challenging situations (Delisle et al. 2016; Zordan et al. 2010), even though the integration of these aspects facilitates the practical implementation of peer support (White et al. 2020). The role of a peer-coach in the clinical context is challenging as the peers provide face-to-face support and share their experience. The coaches should not give any medical or psychological advice but rather reflect their role as a non-professional peer-coach. As their own experience might be different to the paired patient´s experience, they should be able to reflect and discriminate their own cancer history from the patient’s history and experience. All these challenges require – in addition to the preparatory training - a close support and continuous supervision of the peer-coaches through a coordinator.

Although the initial findings show promising effects of peer support in cancer patients, there is a lack of studies that investigate the effects of such innovative programs (Dunn et al. 2003; Weis et al. 2019). In Germany, there are only a few initiatives that systematically include peer support (Salm et al. 2023). The present study was designed to meet the need for research on scientifically evaluated peer support and to satisfy the requirements of the national cancer plan of the Federal Ministry for improving cancer care in Germany. The project relates to the National Cancer Plan of the German Federal Government, which includes patient orientation as a particular field of action and calls for the improvement of patients’ competency through the creation of target-group-oriented, quality-assured and low-threshold service offers, as well as quality-assured counseling and support (Bundesgesundheitsministerium 2019).

The aims of the study were:

1) to develop a peer support program for cancer patients by trained peers.

2) to pilot-test the feasibility, utilization and satisfaction of the program.

3) to investigate the processes and potential outcomes of this peer-coaching program.

## Methods

### Description of the peer-coaching program

In the coaching-program, former cancer patients were trained to empower cancer patients who have a need of social support and to share experiences. The training concept was developed based on a literature search and qualitative semi-structured interviews with patients, patients representatives and experts in psycho-oncology (*n* = 9). After the first run, the concept was slightly adapted based on the results of the evaluation by the trained peer-coaches.

The training of the peer-coaches included 18 teaching units (TU) (TU = 45 min) and was carried out over five half-days covering the following topics: basic information about psychosocial distress, coping with cancer and psycho-oncological care, defining the role of peer-coaching, communication skills, the person-centered attitude according to Carl Rogers, self-care and mindfulness, methods to distance yourself from the suffering of others, resource activation and dealing with difficult conversations. The peer-coaches received regular group supervision and if needed individual supervision under the guidance of a psychologist or psychotherapist/psychooncologist. Due to the SARS-Cov-2 pandemic, two consecutive training groups were carried out online via Zoom between 2021 and 2022. A third training in 2023 was conducted in presence. The evaluation of the training showed that the participants felt well-prepared for their tasks. They also evaluated the online-format of the training as successful, even though some participants noted that they would have preferred to see each other in person.

The trainings were conducted by the project leader (JW: licensed behavioral psychotherapist, psychooncologist) and the study coordinator (AV: psychologist, professional coach) as well as supporting staff. The recruitment of the peer-coaches and patients was carried out by the project coordinator (AV) in cooperation with health professionals specialized in oncological care. Also the screening interviews and the matching between peer-coach and patient were carried out by the project coordinator. Patient and peer-coach had the choice to meet in or outside the university hospital, to talk via telephone or internet. The conversations were free of charge for the patients, the peer-coaches were not remunerated for their engagement in a monetary way.

### Study design and inclusion criteria

This feasibility study was based on a longitudinal observational design with pre-post evaluation of the peer-coaching. To participate in the study as peer-coach, former cancer patients had to meet the following inclusion criteria: Being adult (≥ 18 years), having a history of cancer, completed treatment at least one year ago, mental stability in terms of adaptation to their own cancer experience and sufficient German language skills to follow the training. These criteria were checked in a screening interview prior to inclusion. After inclusion, the volunteers participated in the preparatory training. Potential peer-coaches were recruited through purposive and snowball sampling in the University Hospital of Freiburg, in other regional clinics, through patient organisations and by advertisement in daily newspapers. A total sample of *n* = 25 participants with a cancer history was trained as peer-coaches. The mean age of the peer-coaches was 54.9 years (*SD* = 12.9, range: 28–77 years). More than half of them had a high educational level (university degree 51.5%). The sample was heterogeneous with respect to cancer diagnosis - breast cancer (31%) and leukemia (20.7%) being most frequent - as well as to treatment history and life situation.

Patients with the need to talk to a peer-coach were recruited via flyer, posters, newspaper reports and disseminators at the University Hospital of Freiburg (doctors, psychooncologists, nurses, social worker etc.), rehabilitation clinics and private practices of specialists in the region. All patients with a cancer diagnosis and an interest in peer-coaching were eligible for the study. Before inclusion, interested patients were informed about the study in a short phone call from the program coordinator. After informed consent, we assessed sociodemographic variables such as diagnosis, type of treatment, age and gender as well as the patient’s wishes and expectations towards the program, in order to achieve an optimal fit between patients and eligible peer-coaches. Interested patients were asked in a screening interview with the coordinator about their wishes and preferences concerning their peer-coach. If they did not have any preferences, the coordinator matched the patient according to the following characteristics: diagnosis, treatment history, gender, age or life situation. The coordinator informed the peer-coach with the best fit about the request and initiated the peer-coach to contact the patient for the first conversation. The peer-coach received relevant information about the patient such as age, diagnosis, treatment and concerns as well as the patients’ expectations towards the peer-coaching. Peer-coach and patient decided individually the format (via phone, in presence or online) of the conversation. The peer-coach informed the coordinator about the date of the first appointment and if needed additional appointments. Based on this information the coordinator sent the questionnaires to the patient after the first conversation. We did not define a maximum number of conversations, but the majority used one appointment. After the first conversation, a few patients wanted to keep the option of further appointments with the peer-coach.

### Measures

In a short screening interview with the patient prior to inclusion, the project coordinator informed about the program and collected relevant information like wishes concerning the peer-coach for the best fit between patient and peer-coach. At Baseline, the patients received a set of questionnaires containing the depression module of the Patient Health Questionnaire (PHQ-9) (Kroenke et al. 2001) to measure depression scores, the Generalized Anxiety Disorder 7 (GAD-7) to assess the anxiety level (Spitzer et al. 2006) and the NCCN-distress-thermometer (DT) (Mehnert et al. 2006) to capture psychological distess. The DT includes a single item measuring the subjective level of psychosocial distress (0–10) and a list of 40 issues (yes or no). For descriptive reasons, patients´ social support was assessed only at baseline via the SSUK-8 (Ullrich and Mehnert 2010). Additionally, the prior and actual use of psychological support and other self-help offers, as well as expectations about the peer-coaching were assessed by a self-developed questionnaire at t0. After the first conversation (t1), all patients received a self-developed questionnaire for evaluation of the conversation in terms of experiences and satisfaction (EESC-Pat) together with the standardized questionnaires PHQ9, GAD7 and DT. The peer-coaches were asked about their experiences and satisfaction with the conversation (EESC-Coach) after the first conversation based on an adapted version of the EESC-Pat.

Patients were asked in the t1 questionnaire whether they wanted another meeting with the peer-coach or not. After the last conversation, a final questionnaire was sent to the patients to assess their overall satisfaction with the program. The t2 questionnaire was sent to the patient between three and six months after the last conversation. In cases where patients wanted to keep the option for further appointments, the coordinator proactively contacted the peer-coach and sent the t2 questionnaire to the patient in the same time distance.

The recruitment period lasted from July 2021 until November 2023.

**Fig. 1:**
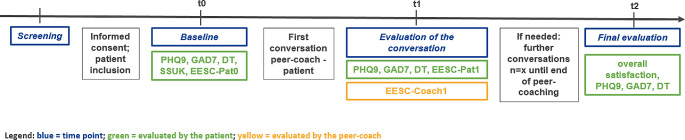
Methods and time points of measurement

In addition, sociodemographic and medical data were collected by the subjective assessment of the patients at t0. Figure 1 shows an overview of the methods and the time points of measurement.

Depending on patients` preferences, they had the possibility to complete the questionnaires either online (LimeSurvey) or in a paper-pencil version. The peer-coaches all completed the questionnaires in the digital way. The data analysis was conducted via SPSS (V29). Primarily descriptive analyses were performed (frequencies, mean and standard deviation). For testing differences between subgroups or time points t-tests were used.

## Results

### Description of sample

**Fig. 2:**
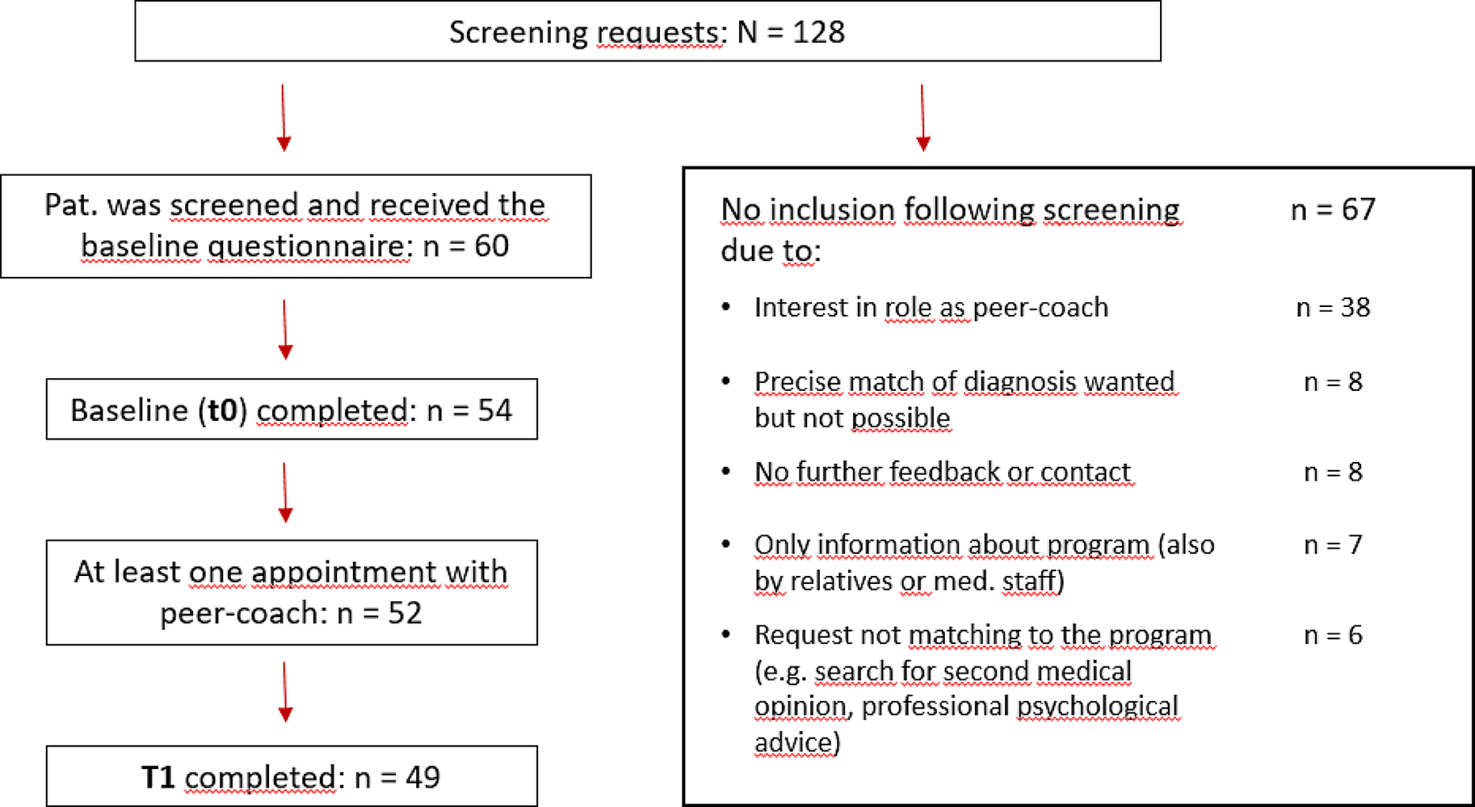
Flow chart of patient inclusion

The coordinator received 127 requests from patients, of which *n* = 60 patients were interested to talk to a peer-coach and received the baseline questionnaire. The most common reasons for non-participation (*n* = 67) are stated in Fig. 2. *N* = 54 patients completed the baseline questionnaire. The final sample for analyses included *N* = 49 patients that had at least one conversation with a peer-coach and a completed questionnaire. The most probable time of patient drop-out in our sample was in the phase of initial diagnosis and ongoing treatment. Figure 2 shows the flow chart of patient inclusion.

The age of the patients at baseline ranged from 27 to 82 years (*M* = 58.04 years; *SD* = 12.63). Most of them were female (77.6%; *n* = 38), only 22.4% (*n* = 11) were male. The majority of the interested patients had a university degree (51.0%). Almost half of the patients was married (49.0%). Most patients were affected by breast cancer (36.7%), while leukemia was the second most common cancer entity (20.4%) in our sample. Apart from that, patients with very different cancer entities were interested in the conversation offer. Most of them had the initial diagnosis (70.8%), only 29.2% were in the phase of cancer recurrence. 56.3% of the participating patients were in the stage of acute treatment while 43.8% had no actual medical treatment. Table 1 shows the sociodemographic and medical characteristics of the patients before baseline.

**Table 1:** Sociodemographic and medical characteristics of the patients before baseline

*n* = 49		
Age (M/SD)	58.04	12.6
	*Frequency*	*%*
**Gender**		
Female	38	77.6
Male	11	22.4
**Marital status**		
Married	24	49.0
Single	9	18.4
Divorced	6	12.2
Separated	5	10.2
Widowed	3	6.1
Committed relationship	2	4.1
**Highest educational level**		
University level	25	51.0
Middle school level	13	26.5
High school level	4	8.2
Secondary school level	4	8.2
Technical college level	2	4.1
Other	1	2.0
**Type of Diagnosis**		
Breast cancer	18	36.7
Leukemia	10	20.4
Skin cancer	4	8.2
Lymphoma	4	8.2
Colorectal cancer	3	6.1
Lung cancer	2	4.1
Brain tumor	2	4.1
Other tumors (each *n* = 1)	6	12.0
**Status of cancer diagnosis**		
Initial diagnosis	34	70.8
Relapse	14	29.2
**Treatment status**		
Ongoing	27	56.3
Completed	21	43.8

### Psychological well-being

The mean value on the Patient Health Questionnaire (PHQ-9), assessing depressive mood, was 9.07 (*SD* = 4.87) which is slightly under the threshold value ≥ 10 indicating a moderate depression, thus a tendency for an increased psychological burden can be seen in the sample. The mean of the anxiety level (GAD-7) of 6.74 (*SD* = 4.19) ranked under the cut-off value for anxiety (≥ 10). The psychosocial distress of the patients, measured via NCCN distress thermometer, had a mean value of 6.36 (*SD* = 2.07). Scores ≥ 5 indicate a higher level of distress and a need for psychosocial support. The results indicate a high subclinical burden and a low level of psychiatric comorbidities in the patients. Regarding the values of the SSUK-8, the positive social support was rather high while detrimental interactions were low. Table 2 shows the psychological parameters of the participants at baseline.

**Table 2:** Psychological parameters of the participants (*n* = 45) at baseline

	M	SD
NCCN-DT ^a^ (sum score)	6.36	2.07
PHQ-9 ^b^ (sum score)	9.07	4.87
GAD-7 ^c^ (sum score)	6.74	4.19
SSUK-8 ^d^ (sum score) Subscale positive support Subscale detrimental interaction	11.57 4.44	3.03 2.61

*Notes* mean scores of participants at baseline on the standardized questionnaires

*M* = mean, *SD* = standard deviation

^a^ DT: *Distress-Thermometer* (Mehnert et al. 2006), Range from 0–10

^b^ PHQ-9: *Patient Health Questionnaire* (Kroenke et al. 2001), Range from 0–27

^c^ GAD-7: *Generalized Anxiety Disorder Scale* (Spitzer et al. 2006), Range from 0–21

^d^ SSUK-8: Questionnaire for social support during illness (Ullrich and Mehnert 2010), Range from 0–16

At baseline, in the problem checklist of the NCCN distress-thermometer the most frequent responses were emotional problems e.g. fears (80.4%), worries (78.3%) and sadness (67.4%). The most common physical problems were exhaustion (82.6%), memory/concentration problems (56.5%) and sleep problems (55.6). Problems in the family, practical problems as well as spiritual concerns were relatively rare.

Only 29.8% of the patients had already joined other self-help offers and only 36.2% actually received professional psychooncological support. In the observational study design, the pre-post comparison of the parameters of the psychological well-being (PHQ-9, GAD-7, DT) was conducted only for heuristic reasons. For the final measurement, we received response only from *n* = 32 patients. Our pre-post comparison showed a significant decline in the stress level (DT) (t(31) = 2.53; *p* = .02) but no significant differences on the PHQ-9 (depressive symptoms) (t(31) = 0.05; *p* = .96) and the GAD-7 (anxiety) (t(31) = 0.42; *p* = .68) between t0 and t1.

### Expectations of the peer-coaching program

The baseline questionnaire comprising the patients’ expectations of the peer-coaching allowed multiple responses. Most frequently, patients expected an exchange of experiences (89.1%), followed by the wish of encouragement (71.7%) and practical information for daily living (69.6%). In addition, talking about their worries was expected (60.9%), whereas support in understanding disease- and treatment-related information (23.9%) was less expected by the patients.

### Evaluation of the conversations with peers

The majority of the 52 patients had one conversation with a peer-coach (71.2%) and one quarter of the patients had 2–4 conversations. A total of 85 conversations were conducted between peer-coach and patient. Table 3 shows the amount and type of conversations.

**Table 3:** Amount and type of conversations

Number of conversations (*n* = 85)	Number of patients (*n* = 52) (%)
1 conversation	37 (71.2)
2–4 conversations	13 (25.0)
≥5 conversations	2 (4.8)
Type of conversation between patient and peer-coach (*n* = 83)	Frequency (%)
telephone	46 (55.4)
in person outside of the clinic	29 (34.9)
in person inside the clinic	7 (8.4)
online	1 (1.3)

Most of the conversations were held via telephone (55.4%) followed by personal appointments outside of the hospital (34.9%). Only a few meetings were conducted personally in the hospital (8.4%) and even less meetings online (1.3%). The average duration of the conversations was about 68.82 min (SD = 28.47) with the shortest talk taking 20 min and the longest one about three hours. Only a few disruptions during the conversations occurred. Technical problems as well as a phone bell ringing or a too noisy atmosphere in a café were mentioned. The patients generally expressed their satisfaction with the organization of the first contact with the coordinator and the peer-coach. They mostly did not have any problems filling out the questionnaires. Overall,the patients rated the contact with the peer-coach positively (see Table 4).

**Table 4:** Evaluation of the conversations at t1 (*n* = 49)

How did you experience the conversation?		M	SD
1	I had the feeling of being understood by the peer-coach.	4.74	0.49
2	In the conversation I was able to address my concerns.	4.67	0.60
3	The conversation strengthened my feeling of not being alone with the situation of the disease.	4.67	0.56
4	The conversation has met my expectations.	4.55	0.58
5	I feel encouraged by the conversation.	4.35	0.70
6	The conversation made me feel more confident.	4.26	0.77
7	The conversation has encouraged me to seek further help.	4.07	1.14
8	The conversation helped me to see more clearly what I want.	3.78	0.96
9	I have received practical everyday tips in the context of the disease.	3.73	1.07
10	The conversation helped me to deal with my anxiety.	3.70	0.94
11	I have received tips where to get more information.	3.67	1.23
12	The conversation helped me to better understand information about the disease.	3.18	1.30
13	The conversation has burdened me.	1.94	0.94
14	The conversation made me feel insecure.	1.30	0.78

*Note* 5-point Likert Scale with a *range* of 1–5 (1 = not applicable at all, 2 = rather not applicable, 3 = partly applies, 4 = rather applies, 5 = fully applies)

The majority of the patients felt able to talk about their concerns with their peer-coach and felt understood by him or her. Most of them felt less alone with the disease due to the conversation. In general, the conversations were not perceived as burdening. Likewise, the patients did not feel insecure after the conversation. Some patients felt encouraged to seek further help. Moreover, some patients felt better in managing their fears. Most patients (94.5%) stated that their expectations were rather or fully met by the conversation. Most of the patients which completed the final evaluation (*n* = 32) affirmed they would recommend the peer-coaching to other affected individuals and considered the offer as an important addition to support offers carried out by professionals. They judged the conversation(s) to be helpful and overall did not feel strained by the conversations. The majority of them was glad they had participated. The satisfaction with the process and effort was also obvious.

### How the peer-coaches experienced the appointments

The peer-coaches received a questionnaire after each conversation with a patient. In total, *n* = 80 questionnaires could be analysed. The coaches were asked to evaluate the conversations on a 5-point Likert Scale (1 = not applicable at all, 5 = fully applies). According to the questionnaires, the peer-coaches usually felt confident in the conversation (*M* = 4.70, *SD* = 0.49) and also felt that they had succeeded in establishing a good contact with the patient (*M* = 4.60, *SD* = 0.61). They mostly experienced the conversation as enriching (*M* = 4.16, *SD* = 1.02). In contrast to that, they did not experience a lot of challenging situations during the conversation (*M* = 1.82, *SD* = 0.94) or perceive the conversation as burdensome (*M* = 1.39, *SD* = 0.63). Only a few situations occured in which the peer-coaches felt challenged: a too quiet or unclear voice of the patient due to the weakening medical treatment, unsupportive reactions of relatives as topic, the progression of the disease or bad news concerning patient´s medical result, an overload of emotional unease as well as questions about disease-specific details when having a different cancer entity.

For the t2 assessment we received response from only *n* = 31 patients. Most of them recommended the peer-coaching to other patients and considered the program as important addition to professional support. Overall, they judged the conversation(s) as helpful and did not feel strained by the communication with the peer-coach. The majority of them appreciated the participation in the program. They were also satisfied with the organisational framework of the peer-coaching. The pre-post comparison of the parameters of the psychological well-being (PHQ9, GAD7, DT) was conducted only for heuristic reasons. There was a significant decline in the stress level (DT) (t(31) = 2.53; *p* = .02) but no significant differences in the PHQ9 (depressive symptoms) (t(31) = 0.05; *p* = .96) and the GAD7 (anxiety) (t(31) = 0.42; *p* = .68).

## Discussion

The peer-coaching is an innovative approach with trained peers providing psychosocial support for cancer patients in the acute situation or in rehabilitation. Patient-engagement is an effective and important social force in medical care, making peer-coaching an important field in future research (Giese-Davis et al. 2006). Our study found a good feasibility of the training and a surprisingly high motivation of former cancer patients to participate in the training and volunteer as peer-coach, even over a long period of time. Many of the peer-coaches described their altruistic motivation to help others by having wished for a program like this when they were in the acute phase of their own disease. The high amount of volunteers is promising to ensure the feasibility of the program. According to the evaluation, the peer-coaches experienced the appointments and their voluntary engagement mostly as enriching and did not feel overstrained by the task.

The patients using the peer-coaching were very satisfied with the program and rated it as helpful and empowering. The results are in line with existing international research on peer support of cancer patients, which found an overall positive benefit for the patients and suggests that participating patients experience positive benefits such as encouragement, an increase of hope and self-confidence (Allicock et al. 2012; Lee et al. 2013; Meyer et al. 2015; Ramchand et al. 2017; Slesina et al. 2014). Considering the sample, more women than men used this program. Moreover, the users had a high level of education. Most of the patients contacted us in the phase of initial diagnosis, where the need of information is higher than in other phases of the disease. We saw the importance of a precise matching between patient and peer-coach concerning the diagnosis, which is consistent with the results of corresponding studies (Prashar et al. 2022; Rudy et al. 2001). Overall, our study results are promising as they show a high goal attainment and a great potential to support cancer patients by peers in addition to professional services. The organisational structure of the program turned out to be helpful for the patients and the peer-coaches. We want to point out, that the coordinator plays an important role for the success of the program.

The collaborating clinic departments and rehab centers were open to the idea of integrating the peer-coaches as a non-professional support option and an additional program for patients. They considered it an important offer to improve the psychosocial support for cancer patients. The project coordinator was important for the organization and structure of the study and served as a central contact person for the patients, peer-coaches and multiplicators. In particular, the principle of patient participation, which is increasingly important in health policy, was successfully implemented in the project by including patients’ representatives in the development of the program and all study materials. In spite of these positive findings, the number of patients that used the peer-coaching was smaller than expected. This might be due to an overload of psychosocial care in the hospitals in addition to the existing services of social and psychooncological care. High distress before, during and immediately after the treatment, an overload of challenges and decision making in terms of treatment or an uncertainty about the potential benefit of peer-coaching in this period might also be considered as reasons for this result. Unfortunately, the start of our peer-coaching program was during the high phase of the Coronavirus pandemic which might also have influenced the acceptance of a new program. To clearly understand the reasons for the restrained demand of the program, a strengths-and-weaknesses analysis is needed, also to clearify the cost-benefit ratio of the approach. Due to the small sample size, more research is needed to verify the tendencies shown in our feasibility study. For further research, a controlled design should be put into practice to examine the effects of the program with respect to quality of life and other parameters such as depression, anxiety and psychosocial distress.

### Study limitations

As already mentioned in the discussion, our peer-coaching program and in particular the recruitment phase during 2021 and 2022 was strongly influenced by the Coronavirus pandemic, in which the vulnerable group of cancer patients limited their social contacts and clinic appointments. This circumstance had a negative impact on the amount of patients that received the information about the program and participated in the study by talking to a peer-coach. Apart from these obvious challenges, also in 2023 the user rates were lower than expected and the sample size of the pre-post comparison in our study is too small to draw a conclusion whether or not the program has a significant positive impact on the psychological well-being of the patients. Additionally, in the t2 follow-up we had a very low return rate. Therefore, the results are preliminary based only on a small sample. As a feasibility study we tested the program only in a limited regional area focusing on the university hospital.

### Clinical implications

One-to-one-peer support with trained and regularly supervised peer-coaches is a promising offer for cancer patients in addition to professional psychooncological care. The clinical experts were open to integrate the offer in their clinical praxis, but the implementation of an innovative approach needs time to be accepted by patients and the professional staff. Although the peer-coaches act as volunteers, the indispensable coordination of the program by a project coordinator needs financial resources.

## Conclusion

Based on our experiences and the preliminary positive results, we plan a multicentre study with a control group in collaboration with other clinic centers across Germany to achieve a larger sample size.

## Data Availability

Generated datasets are not publicly available due to privacy concerns. The data can be obtained by the corresponding author upon reasonable request.
